# Towards the automation of NIR spectroscopy to assess vineyard water status spatial–temporal variability from a ground moving vehicle

**DOI:** 10.1038/s41598-023-39039-z

**Published:** 2023-08-17

**Authors:** Juan Fernández-Novales, Ignacio Barrio, María Paz Diago

**Affiliations:** 1 Department of Agriculture and Food Science, University of La Rioja, 26007 Logroño, La Rioja Spain; 2 Institute of Grapevine and Wine Sciences, University of La Rioja, Consejo Superior de Investigaciones Científicas, Gobierno de La Rioja, 26007 Logroño, La Rioja Spain

**Keywords:** Plant stress responses, Infrared spectroscopy

## Abstract

Irrigation has a strong impact in terms of yield regulation and grape and wine quality, so the implementation of precision watering systems would facilitate the decision-making process about the water use efficiency and the irrigation scheduling in viticulture. The objectives of this work were two-fold. On one hand, to compare and assess grapevine water status using two different spectral devices assembled in a mobile platform and to evaluate their capability to map the spatial variability of the plant water status in two commercial vineyards from July to early October in season 2021, and secondly to develop an algorithm capable of automate the spectral acquisition process using one of the two spectral sensors previously tested. Contemporarily to the spectral measurements collected from the ground vehicle at solar noon, stem water potential (Ψ_s_) was used as the reference method to evaluate the grapevine water status. Calibration and prediction models for grapevine water status assessment were performed using the Partial least squares (PLS) regression and the Variable Importance in the Projection (VIP) method. The best regression models returned a determination coefficient for cross validation (R^2^_cv_) and external validation (R^2^_p_) of 0.70 and 0.75 respectively, and the standard error of cross validation (RMSECV) values were lower than 0.105 MPa and 0.128 MPa for Tempranillo and Graciano varieties using a more expensive and heavier near-infrared (NIR) spectrometer (spectral range 1200–2100 nm). Remarkable models were also built with the miniaturized, low-cost spectral sensor (operating between 900–1860 nm) ranging from 0.69 to 0.71 for R^2^_cv_, around 0.74 in both varieties for R^2^_p_ and the RMSECV values were below 0.157 MPa, while the RMSEP values did not exceed 0.151 MPa in both commercial vineyards. This work also includes the development of a software which automates data acquisition and allows faster (up to 40% of time saving in the field) and more efficient deployment of the developed algorithm. The encouraging results presented in this work demonstrate the great potential of this methodology to assess the water status of the vineyard and estimate its spatial variability in different commercial vineyards, providing useful information for better irrigation scheduling.

## Introduction

In the actual context of climate change water use is becoming a critical issue in sustainable viticulture since periods of strong variability and uncertainty in water resources availability are forecast^1^. At the same time, in many regions, water quality is decreasing because of salination caused by excessive water uptake and depletion in rivers and basins and water rising sea levels, as well as of the increase of polluting activities.

In viticulture, irrigation has a strong impact in terms of yield regulation and grape and wine quality, so the implementation of precision watering systems would facilitate the decision-making process about the water use efficiency and the irrigation scheduling in viticulture^2^. In this framework, the usefulness of high-spatial resolution information concerning plant water status within-plots has been reported^3,4^ with the intention to provide grapevines under different water requirements with different irrigation doses, that is smart irrigation. Hence, smart irrigation strategies emerge as a potential solution to optimize vineyard water usage with the aim to protect grapevines from severe water deficit stress.

Conventional approaches to appraise the plant water status range from soil water measurements to environmental modelling and plant-based methods^5^. Nevertheless, most of these methods, although informative, are either destructive, laborious and unsuitable to characterize the vineyard spatial variability^6^. To prevent most of the pitfalls of classical plant-based methods, other technologies linked to remote and proximal sensing, which record information about plants are being developed. The two main non-destructive technologies employed in the assessment of grapevine water status and its corresponding vineyard spatial variability are thermography and VIS–NIR spectroscopy. In both cases, information and data gathered with the sensors are then validated against a plant-based water status reference method, generally plant water potential (Ψ) or stomatal conductance (g_s_)^7,8^.

Over the last decade, thermal cameras of different resolution and prices used as portable devices^9,10^ or-mounted on ground^7,11^ and unmanned aerial vehicles^8,12–14^ have been used to monitor canopy temperature in grapevines. Nevertheless, the general use of reference temperatures^7^ in thermography applications somehow has hindered its extensive usage in commercial operations.

NIR spectroscopy is a technique that provides rapid and non-destructive data acquisition, easy usage, and little sample preparation, which has enabled to monitor plant water status at leaf level in various species^15,16^ including grapevines, using different portable devices under field conditions with a good performance^17–20^. Water is a major component of grapevine leaves. In the NIR range (from 800 to 2500 nm), the O–H second overtone is shown around 978 nm, the O–H stretch first overtone at 1454 nm, and the combination bands of O–H bonds in hydroxyl groups show up around 1930–1940 nm^21^. The reduced number of representative measurements scanned with portable spectral devices to evaluate the spatial variability of the vineyard, together with the need to automate the process encouraged the development of manned or autonomous mobile sensing platforms for assessing and mapping vineyard water status in the last years^8,22^.

Nowadays, NIR spectral devices mounted on ground vehicles have proven to yield good performance in terms of determination coefficient of prediction (R^2^_P_ ~ 0.68–0.85) and root mean square error (RMSEP ~ 0.131–0.190 MPa) for predicting plant water status^8,22^. However, these sensors are expensive, heavy and require a lot of space when assembled in a mobile platform, so it would be necessary to move towards more compact, miniaturized spectral devices, of lower cost, that enable the acquisition of spectral information, in an easier and more affordable way. Moreover, as pointed out by Pampuri et al.^20^, currently, no cost-effective commercial stand-alone devices capable of rapidly estimating water status directly in the field in an automated way are available on the market and this was highlighted as one of the major weaknesses of the use of NIR spectral data in the viticulture sector. However, optics and electronics grow and develop faster than ever. Likewise, new, smaller and less expensive spectral devices emerge every year and the challenge of applying this technology to be used directly in the field to help the small grower optimizing water use efficiency in viticulture is getting closer. Furthermore, the NIR approach to assess grapevine water status offers two main opportunities^20^ in their strength-weakness-opportunity-threat (SWOT) analysis: to monitor crop water status in a semi-continuous way, and to improve irrigation efficiency. The former could upgrade to continuous monitoring should the process is further automated, while the latter is certainly relevant in the actual context of climate change where uneven water availability and increased drought periods are experienced, thus requiring a more optimized water usage.

Under this scenario, this work aims at contributing to the development of NIR spectroscopy as an operational method to assess vineyard water status in commercial premises by focusing on two main goals. The first was to test and compare two NIR spectral devices, one of them being a new, low-cost, miniaturized NIR spectral sensor, for the assessment and mapping of grapevine water status from a ground moving vehicle in two commercial vineyards. The second goal was to develop an algorithm capable of automate the spectral acquisition process in the field to increase its efficiency, rapidness and robustness.

## Materials and methods

In order to address the first goal, the experiment was split into three related tasks: first, to assess grapevine water status using a new, fast, miniaturized, and low-cost NIR spectral sensor installed in a ground vehicle, collecting data from two different commercial vineyards; second, to evaluate and compare the applicability of this low-cost spectral sensor facing another more robust and expensive NIR device; third, to monitor and map the spatiotemporal evolution of the vineyards’ water status along season 2021 with both devices. Regarding the second goal, the development of the algorithm was focused on the automation of the spectral measurement with the miniaturized NIR device. All the methods were carried out in accordance to relevant Institutional guidelines and regulations.

### Vineyard site and experimental layout

The experiment was carried out in two commercial vineyards located in Tudelilla, La Rioja, Spain during the summer months of July, August, September and October 2021, along the grapevine ripening period. Permissions to carry out the experiments in the two plots were granted to the researchers of this study by the viticulture manager and owner of the vineyards at Bodegas Vivanco (https://vivancoculturadevino.es/es/bodega/terrunos-y-variedades/). The Tempranillo (*Vitis vinifera* L.) vineyard was planted in 2002 following a north–south orientation (Lat. 42°,18′18.26″, Long. − 2°,7′14.15″, Alt. 515 m) while the Graciano (*Vitis vinifera* L.) vineyard was planted in 2016 following a northwest-southeast orientation (Lat. 42°18′30.52″, Long. − 2°7′05.17″, Alt. 497 m). Both grapevine varieties were trained to a vertically shoot-positioned trellis system on a double-cordon Royat and grafted on rootstock R-110 with vine spacing of 2.60 m between rows and 1.20 m between vines.

**Figure 1:**
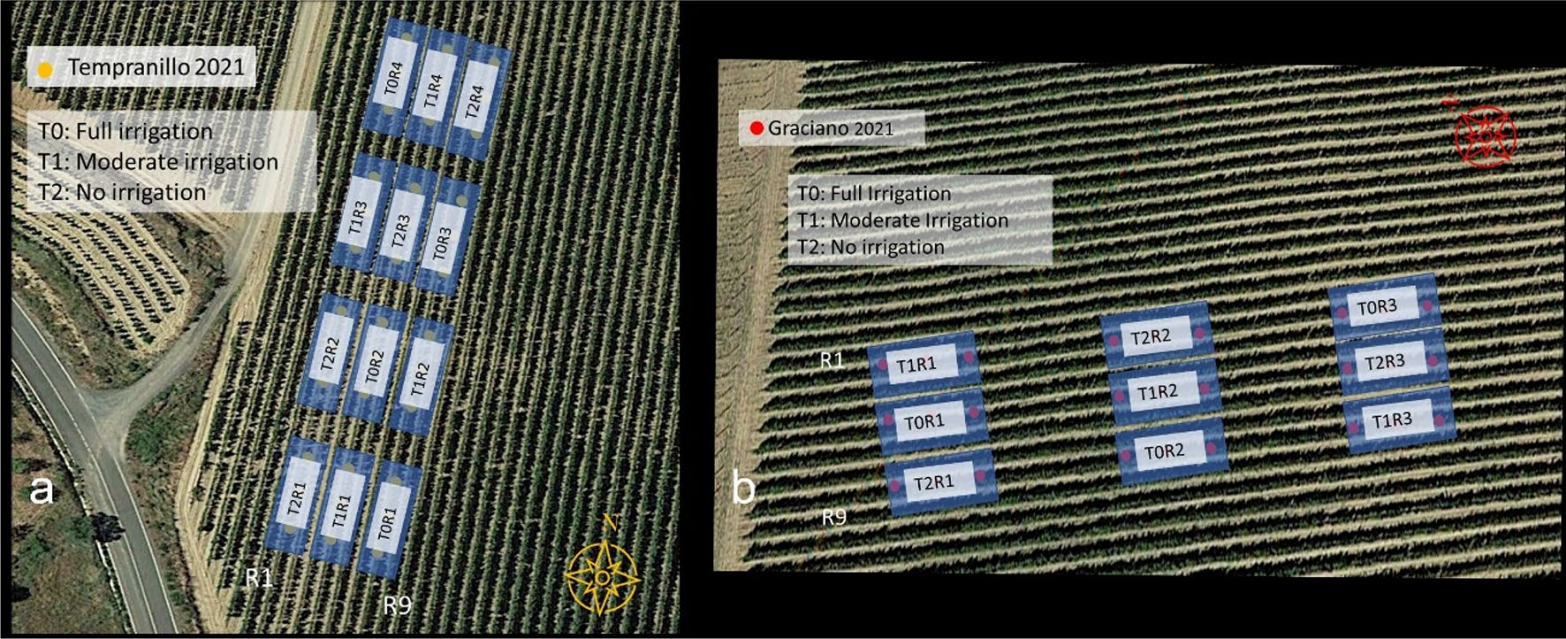
Experimental layout resulting a completely randomized block design with four replicates in (**a**) a Tempranillo vineyard plot, and (**b**) three replicates in a Graciano vineyard plot during season 2021. (This figure was prepared with QGIs 3.28.8 Firenze (https://qgis.org/en/site/forusers/download.html) using imagery available at IDE Rioja (https://www.iderioja.larioja.org/index.php?id=8&lang=es), with open access license CC BY).

In order to create a wide variability of grapevine water status, three different water regimes were established in a completely randomized block design (Hinkelmann and Kempthorne 2007^23^) in each vineyard plot. The irrigation treatments were as follows: T0, full irrigation. Two water pipelines irrigating (6 L h^−1^) were installed, applying two hours per day, five days a week; T1, moderate irrigation. A single water pipeline irrigating half the amount in T0 was installed, and T2, no irrigation. The Tempranillo vineyard plot was designed with four replicates per treatment, making a total of 12 replicates (Fig. 1a), while Graciano vineyard was deployed with only three replicates, nine blocks in total because of the reduced dimension of the plot (Fig. 1b). These replicates were composed by three rows and only the middle row was monitored to avoid the edge effects. Each replicate was represented by three consecutive sections of five plants (15 plants per replicate).

### Sensors and ground vehicle

On-the-go spectral measurements in both vineyards were acquired using two different NIR sensors that were assembled in the ground vehicle to estimate the grapevine water status.

One of these sensors (sensor #1) was the Polytec PSS 2120 spectrometer (Polytec GmbH, Waldbronn, Germany) working in the 1100–2100 nm spectral range, at a 2 nm resolution, with 501 datapoints per spectrum, and 14 Hz acquisition rate. This spectrometer was a NIR optical device (of dimensions 200 × 250 × 135 mm and weight 6.35 kg, not accounting for the non-contact probe, optic fibre and cabling), based on a polychromator as reflection light source selector, and Indium Gallium Arsenide (InGaAs) diode array detectors. This device contains a sensor head (based on an integrated 20 W tungsten halogen lamp) for light capturing, a processing unit and an optical fibre linking them.

Sensor #2 was the Insion 1.7 NT/H spectrometer (Insion Gmbh, Obersulm, Germany). It is a NIR micro spectrometer, which operates in the 900–1860 nm spectral range, at 8.2 nm resolution and 12.5 Hz acquisition rate. This miniaturized low-cost sensor weights 130 g and its dimensions are 108 × 77 × 21 mm. Since this is a passive sensor, the illumination provided by the Polytec PSS 2120 sensor head was used to gather an adequate spectral signal. The signal to noise ratio was preliminary tested for this device using this lamp source.

**Figure 2:**
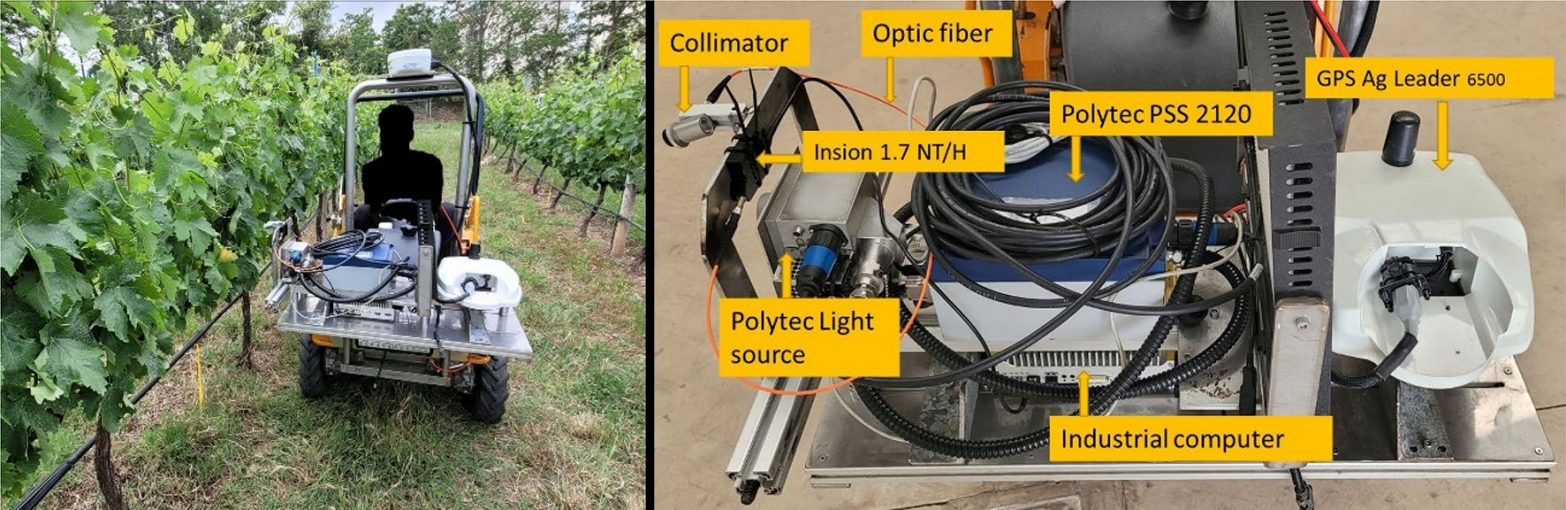
(**a**) Ground vehicle to assess the vineyard water status and (**b**) detail of the instrumentation assembled in the mobile platform for on-the-go spectral measurements in the Tempranillo and Graciano vineyards plots.

These two sensors were installed in a ground vehicle (Fig. 2), which was a modified brushcutter (940 Sherpa 4WD XL, AS-motor, Bühlertann, Germany) capable to make spectral acquisitions controlled by a tablet connected via WIFI to the industrial computer (also installed in the vehicle) and operate simultaneously with both NIR sensors while the ground vehicle was in motion at a constant speed of 3 km h^−1^. The vehicle was also equipped with a RTK GPS receiver (AG Leader 6500 with RTK relay) with centimetric precision and 20 Hz refresh rate, which is connected to the industrial computer with an RS-232 serial connection.

### On the go spectral measurements

Spectral acquisition using the ground vehicle was conducted at solar noon (between 14:00–15:00 GMT + 1) on the east side of the canopy (Tempranillo vineyard plot) and on the northeast side of the canopy (Graciano vineyard plot) from July to early October 2021.

The Polytec sensor head was placed at a height of 1.0 m from the ground, pointed to the canopy on a lateral point of view at 0.30 m of distance (Fig. 2). The NIR micro spectrometer collimator was aligned with the sensor #1 light source to collect the maximum reflected radiation and maintained at the same distance to the canopy. Spectral measurements were georeferenced using a GPS receiver Ag Leader 6500 (Ag Leader Technology, Inc., Ames, IA, USA) with RTK correction installed on the ground vehicle. Sensor #1 was utilized the first four dates of measurement (in the Graciano vineyard) and the first five dates (in the Tempranillo vineyard) only, since an internal component of the spectrophotometer was damaged in mid-August and could not be fixed during the season. On the other hand, spectral measurements with sensor #2 were acquired along six different dates from July to October in both vineyards.

### Development of an algorithm and custom software for automated spectral acquisition with a miniaturized sensor

Since one of the goals of this work was to move forward automation of spectral measurements, an algorithm was developed in order to automate the control of the sensor #2 mounted in the ground vehicle. This piece of software was implemented using C# as the programming language, targeting a good balance between efficiency at run time and relatively short development time. Also, the .NET Framework provides good integration with the rest of the software already installed on the industrial computer. Any future part of the software requiring higher performance could be developed using C++ CLI which would allow reutilization of most of the existing code.

The communication between the computer and the NIR spectral sensor was carried out by a high speed but relatively simple serial connection using USB interface. A software better suited for field usage was developed utilizing both serial monitoring tools and the documentation provided by the manufacturer.

### Measurement of the stem water potential (Ψ_s_)

The reference method used for the measurement of the plant water status was the stem water potential (Ψ_s_). The 15 plants in each replication block were sorted into three groups (5 vines per group). In each group a random vine was marked, and one leaf from the mid-upper part of the canopy was selected and its stem water potential measured using a Schölander pressure bomb (Model 600, PMS Instruments Co., Albany, USA) at the same time as on-the-go spectral measurements were acquired. Therefore, 36 leaves per day were measured in the Tempranillo vineyard plot and 27 leaves per day in the Graciano vineyard plot, making a total of 252 and 189 measurements of Ψ_s_, respectively during season 2021. Prior to the Ψ_s_ measurement, the selected leaves were covered with aluminium foil to drive them into dark adaptation for at least one hour.

### Spectral processing and data analysis

Once the spectral data were acquired with each sensor, the spectral processing consisted of the following steps: allocation of the spectral measurements to the different replication blocks of the experimental tests, spectra comparison and filtering of the measurements acquired in motion against to a grapevine leaf spectral signature collected under the same microclimatic conditions to avoid the presence of gaps, wires, wood, irrigation pipes, grape berries, etc. After the average of the selected grapevine leaf spectra and removal of light scattering effects, they were linked with its corresponding reference of Ψ_s_. The different steps of spectral data processing are further described in^8,22^.

The spectral processing of the data acquired with sensor #1 (Polytec PSS 2120) was performed through the “Spectra Comparison and Filtering” tool from the SL Utilities software (version 3.1, Polytec GmbH, Waldbronn, Germany). Only spectra which passed the “Spectra Comparison and Filtering” thresholds were considered as valid to be used in calculating the average spectrum per block. The settings Cosine was the method used to adjust the threshold value to determine the required similarity of the raw on-the-go spectra to the defined signature of grapevine leaf spectrum. A higher threshold value (close to 1) means that greater similarity is required to accept the measured spectrum as a true grapevine leaf spectrum. The threshold value in the field experiment was set to 0.985. The same spectral handling and threshold value of the setting cosine was applied to the on-the-go spectral measurements collected with the NIR micro spectrometer, sensor #2 through a code programmed in Matlab (version 2019a, The Mathworks Inc., Natick, MA, USA).

Calibration and prediction models for grapevine water status assessment were performed using the PLS Toolbox (version 8.1, Eigenvector Research, Inc., Manson, WA, USA) software in conjunction with MATLAB (version 2019a, The Mathworks Inc., Natick, MA, USA). Partial Least Squares (PLS) regression was used as the algorithm for training the plant water status prediction models. This algorithm has proved to be an accurate, robust, and reliable chemometric method^24^ to analyse spectral data, as it is capable to deal with a vast amount of data, especially when the number of attributes (wavelengths in this case) largely surpasses the number of samples.

A ten-fold Venetian blind method was used for the internal cross validation and to choose the optimal number of latent variables (LV) which was selected as the one yielding the minimum root mean square error of cross validation (RMSECV) as well as to obtain an estimation of the error rate of the models. We refer to the model with the lowest possible number of LV (or PLS factors) until the first minimum value of the error is reached. This might not be the absolute minimum of the error, only the first local minima (Fig. S1).

To evaluate the quality of the models, the determination coefficient of calibration (R^2^_c_) and cross validation (R^2^_cv_), the root mean square error of calibration (RMSEC) and cross validation (RMSECV) were calculated.

For completeness, two models using the same calibration (train) and validation (val) split were generated for each one of the varieties studied in the manuscript, using respectively a Random Forest Regressor and a Multi-layer Perceptron Regressor with data collected with sensor #2. These models were implemented using Python release 3.11 and the latest stable version of the scikit-learn libraries, which are the de facto standard for classical machine learning models.

For the sensor #2 an external validation (also called prediction) was conducted utilizing 20% of the initial dataset for both varieties. The criteria to select the external validation set was to choose seven random samples for each date leaving out the maximum and minimum values of stem water potential for each of the dates in order to minimize the influence of possible outliers. The metrics used to evaluate the predictive capacity of the external validation models were the determination coefficient of prediction (R^2^_p_) and the root mean square error of the prediction (RMSEP).

The Variable Importance in the Projection (VIP) method^24^ was used to identify and evaluate the relative importance of each wavelength in the best PLS models. VIP score values were computed as the explained sum of squares by the PLS dimension, summed for all dimensions related to the total explained sum of squares by the PLS model and for the total number of wavelengths. Since the average of squared VIP scores is equal to 1, influential wavelengths can be considered to be those with VIP scores greater than 1^24^.

### Mapping

Maps of the predicted values of Ψ_s_ obtained with the ground vehicle and maps of the measured values of Ψ_s_ collected with the reference method were created using empirical Spline interpolation (Earls and Dixon 2007^25^), implemented in ArcGis 10.3 (Environmental Systems Research Institute, Redlands, CA, USA). To explore the variability of the plants’ water status, interpolation with no more than five classes was selected, to ease zone segmentation and decision making based on the map, while maintaining reasonable granularity.

### Ethical statement

The authors declare that they have no known competing financial interests or personal relationships that could have appeared to influence the work reported in this paper. The conducted work does not involve animal nor human experimentation of any kind.

## Results and discussion

### Vineyards water status

Table 1 shows the values of range, standard deviation and mean for Ψ_s_ gathered from pre-veraison to harvest for the two grapevine varieties during season 2021.

**Table 1:** Descriptive statistics of the stem water potential (Ψ_s_) data measured in commercial vineyards of Tempranillo and Graciano varieties during season 2021.

Ψ_s_ (MPa)	Tempranillo vineyard					
	Date of measurement					
	7th July	20th July	27th July	11th August	25th August	15th September
# Samples	35	36	36	36	36	36
Maximum	− 0.85	− 1.05	− 1.00	− 1.30	− 1.45	− 1.30
Minimum	− 1.45	− 1.50	− 1.55	− 1.80	− 1.90	− 2.15
SD	0.14	0.10	0.14	0.11	0.15	0.22
Mean	− 1.13	− 1.23	− 1.26	− 1.51	− 1.68	− 1.82

Ψ_s_ (MPa)	Graciano vineyard					
	Date of measurement					
	14th July	21st July	12th August	26th August	8th September	7th October
# Samples	27	27	27	27	27	27
Maximum	− 0.05	− 0.45	− 0.65	− 0.25	− 0.35	− 0.85
Minimum	− 0.60	− 0.70	− 1.20	− 1.20	− 1.00	− 1.35
SD	0.16	0.07	0.14	0.22	0.19	0.16
Mean	− 0.36	− 0.59	− 0.86	− 0.83	− 0.62	− 1.08

The average water stress level trend was much more gradual and severe in the Tempranillo (Ψ_s_ = − 1.13 to − 1.82 MPa) than in the Graciano vineyard plot (Ψ_s_ = − 0.36 to − 1.08 MPa), whose most negative value (Ψ_s_ = − 1.35 MPa) only revealed a moderate stress^26^. The most stressed values for stem water potential were achieved in mid-September (Ψ_s_ = − 2.15 MPa) in the Tempranillo’s plot while the first week of October was for the Graciano’s plot with a value of − 1.08 MPa. The evolution of the stem water potential in the latter was less progressive and it even dropped remarkably on September 8th towards less negative stress values (− 0.62 MPa) possibly due to some rainfall (13.10 l m^−2^) recorded the previous week by a local weather station of the Rioja Government.

Overall, in both vineyards, an adequate range of variation of the plant water status was created along the season, which was necessary to build the spectral-based models.

### Regression models for grapevine water status

**Figure 3:**
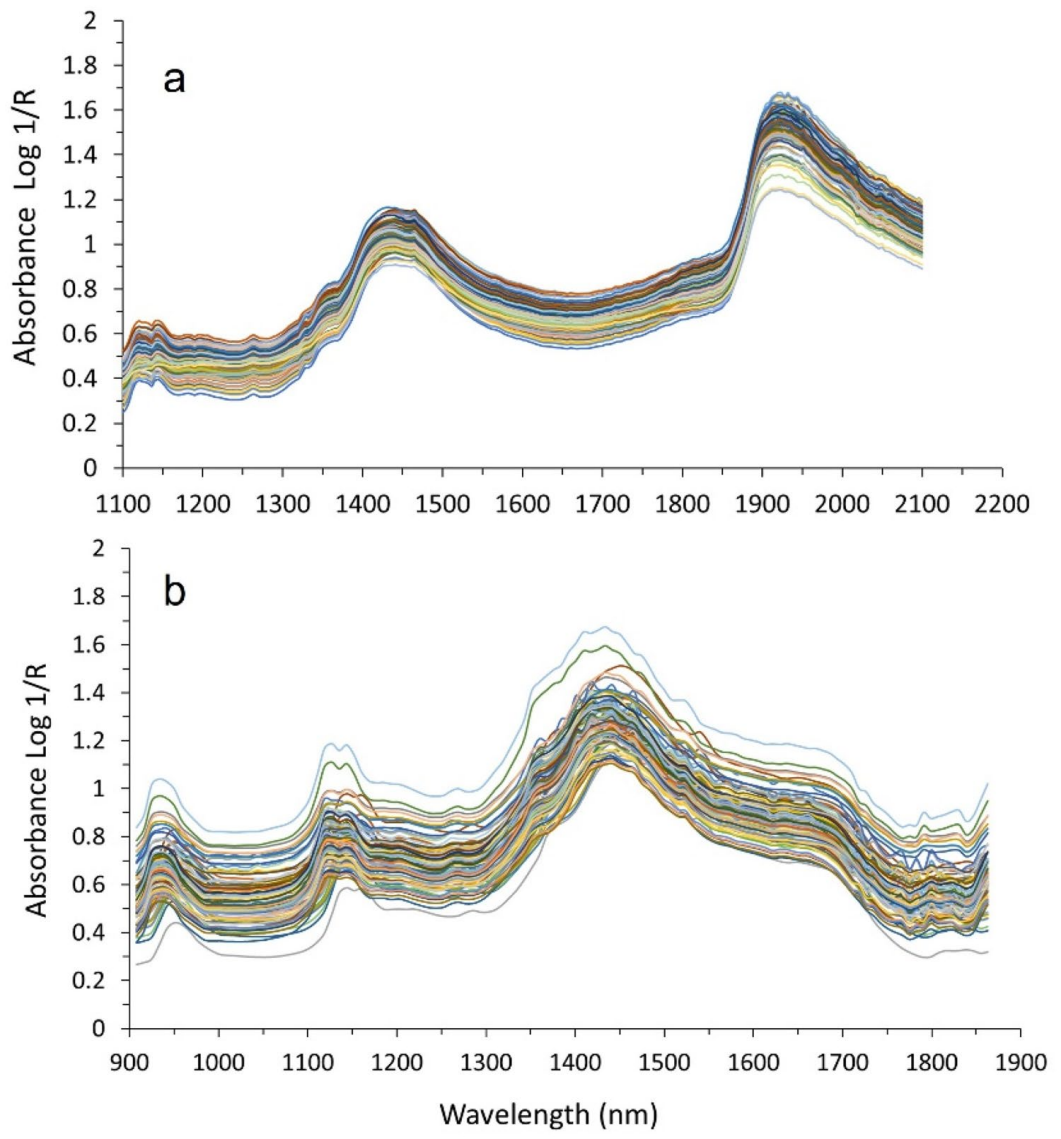
Examples of the whole set of original spectra acquired with (**a**) sensor #1 and (**b**) sensor #2 during a measurement date.

Figure 3a,b show the original spectra for sensor #1 and sensor #2 respectively.

Different combinations of spectral preprocessing operations (smoothing, scattering correction and derivatives) were applied to obtain the models with the best prediction outputs. Likewise, the best PLS regression models included the implementation of the standard normal variate (SNV) together with the Savitzky–Golay first derivative. The best models obtained for Ψ_s_ were selected by statistical criteria, choosing those that yielded the lowest values of RMSECV and highest values for R^2^_cv_ with a lower number of latent variables to avoid overfitting.

#### Regression models using sensor #1

**Figure 4:**
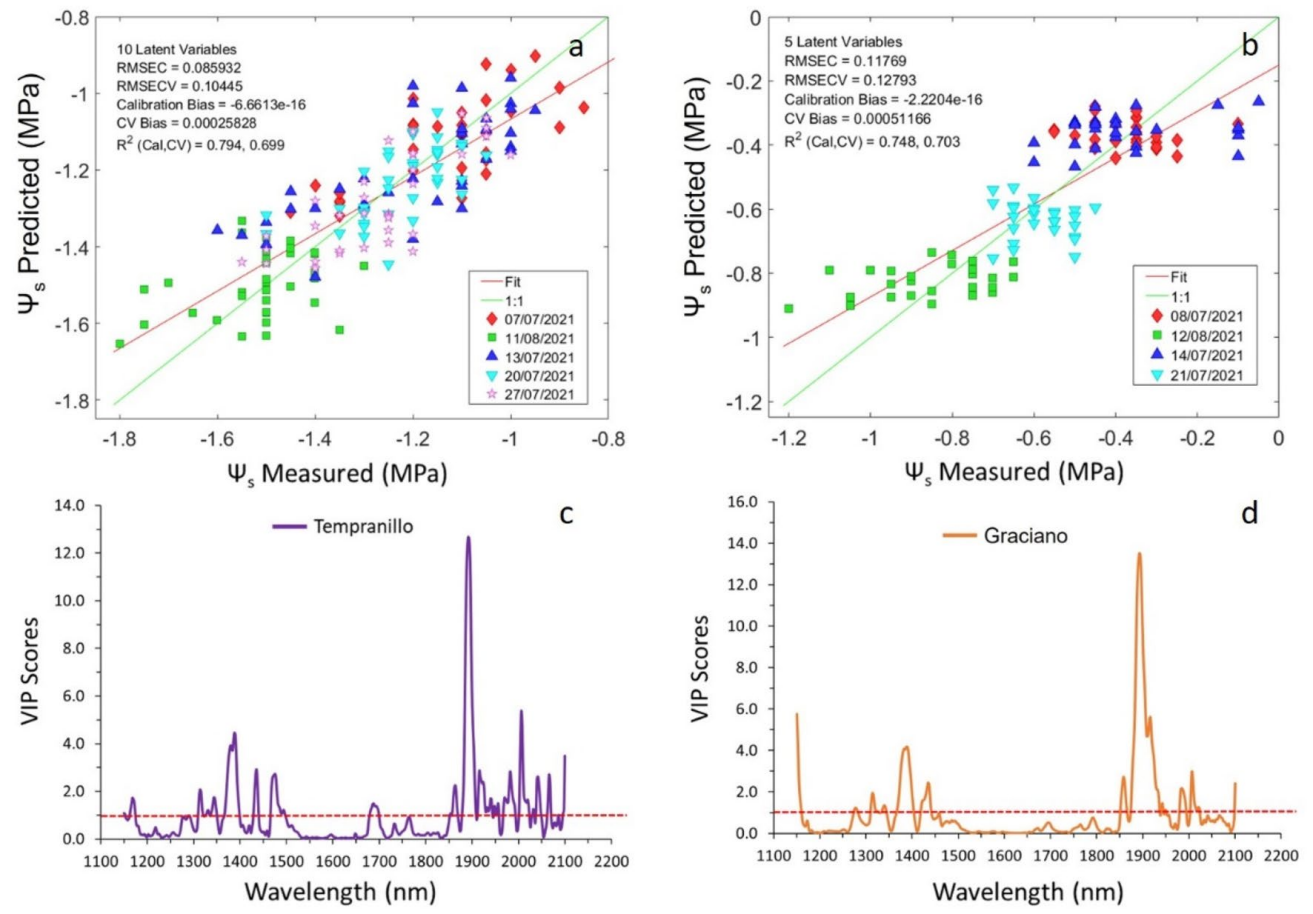
The best PLS regression model for Ψ_s_ prediction using sensor #1 (PSS 2120 spectrometer) in (**a**) a Tempranillo, (**b**) commercial vineyards during 2021 season and corresponding Variable Importance in the Projection (VIP) score value for the PLS models of (**c**) Tempranillo and (**d**) Graciano.

The best regression models for cross validation obtained with sensor #1 returned a determination coefficient value of 0.70 for Ψ_s_ in both Tempranillo and Graciano vineyard plots. The accuracy of the models in terms of RMSECV value was 0.104 MPa for the Tempranillo (Fig. 4a) vineyard plot and 0.128 MPa for Graciano (Fig. 4b).

The VIP scores of the PLS models that correspond to the most influential variables or wavelengths to estimate the stem water potential in both grapevine varieties collected with sensor #1 are shown in Fig. 4c,d, for Tempranillo and Graciano, respectively. The highest VIP score value was located in the 1888–1900 nm region with an absorption peak at 1892 nm with values of 12.68 (Tempranillo) and 13.51 (Graciano). Other relevant regions of the spectrum were those between 1350–1500 and 2000–2100 nm resulting from the first overtone of the O–H bond and the combination band of water, as already revealed in previous works^18,22,27^.

#### Regression models using sensor #2

Random Forest Regressor and Multi-layer Perceptron Regressor models yielded considerably lower metrics according to RMSEP and R^2^ and were more prone to overfitting (Fig. S2). Since the main goal of this manuscript was to test the capabilities of the simpler sensor (sensor #2) versus the more complex spectrometer (sensor #1) and to assess the viability of sensor #2 in a semi-automated way, with the significant reduction in cost and complexity of operation, only the PLS results have been included and discussed in this work, although regressions of the two machine learning-derived models are included as Fig. S2 in the supplementary material section.

**Figure 5:**
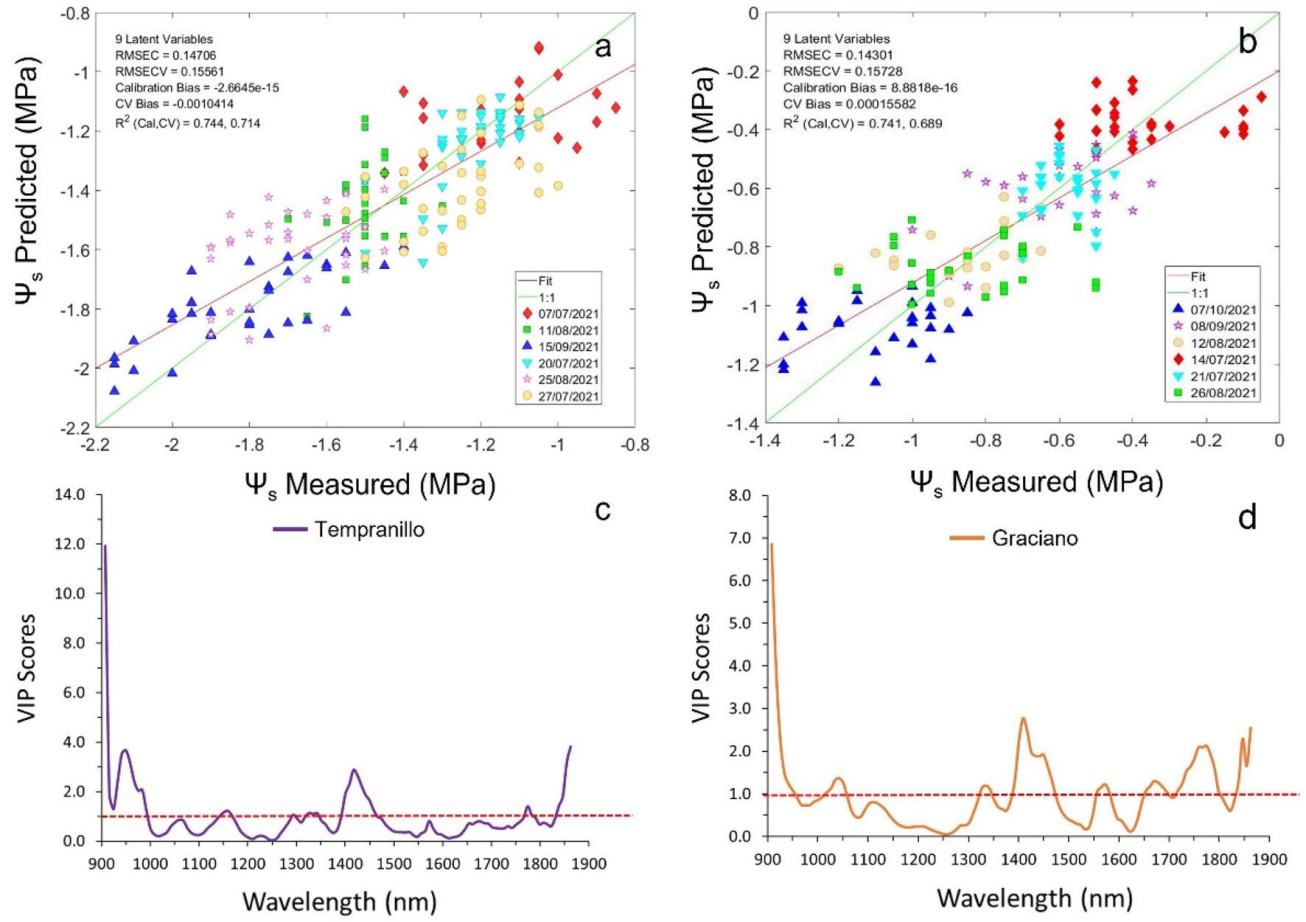
The best PLS regression model for Ψ_s_ prediction using sensor #2 (1.7 NT/H spectrometer) in (**a**) a Tempranillo, (**b**) commercial vineyards during 2021 season and corresponding Variable Importance in the Projection (VIP) score value for the PLS models of (**c**) Tempranillo and (**d**) Graciano.

Figure 5 displays the best PLS models for Ψ_s_ prediction using the micro NIR spectrometer (sensor #2) and their VIP scores projections in the two grapevine varieties.

The results achieved with sensor #2 in terms of R^2^_cv_ were very similar to those returned with sensor #1, showing values of 0.71 for the Tempranillo model (Fig. 5a) and 0.69 for Graciano (Fig. 5b). The accuracy of the models (RMSECV = 0.156 MPa for the Tempranillo variety and RMSECV = 0.157 MPa for Graciano variety) was slightly lower than the one yielded by sensor #1 models (Fig. 4a,b). However, these outcomes are in good agreement with previous works carried out on with sensor #1 installed on a moving all-terrain vehicle that reported a RMSECV = 0.171 MPa in season 2015^8^ and a RMSECV = 0.157 MPa^22^ using in both cases a range of Ψ_s_ from − 2.25 to − 0.55 MPa. Furthermore, the sensitivity of the models generated with this miniaturized, and low-cost spectral sensor (sensor #2) assembled in a ground vehicle was similar or superior to that yielded in other studies with portable hand-held sensors, which ranged between 0.160 and 0.180 MPa^17,19^.

Regarding the VIP scores’ projections for sensor #2-built PLS models (Fig. 5c,d), it can be seen that the most important regions are those between 900–1000, 1400–1500 and 1850–1900 with scores at their maximum peaks for the Tempranillo and Graciano models of 11.9 and 6.85 (907.94 nm band), 2.9 and 2.76 (1417.29 nm band), and 3.8 and 2.54 for the 1862.85 nm band. As in the models built using sensor #1 (Fig. 4c,d), these regions are directly related to the overtones of the O–H bond (first and second) and the combination band of water^28^.

**Figure 6:**
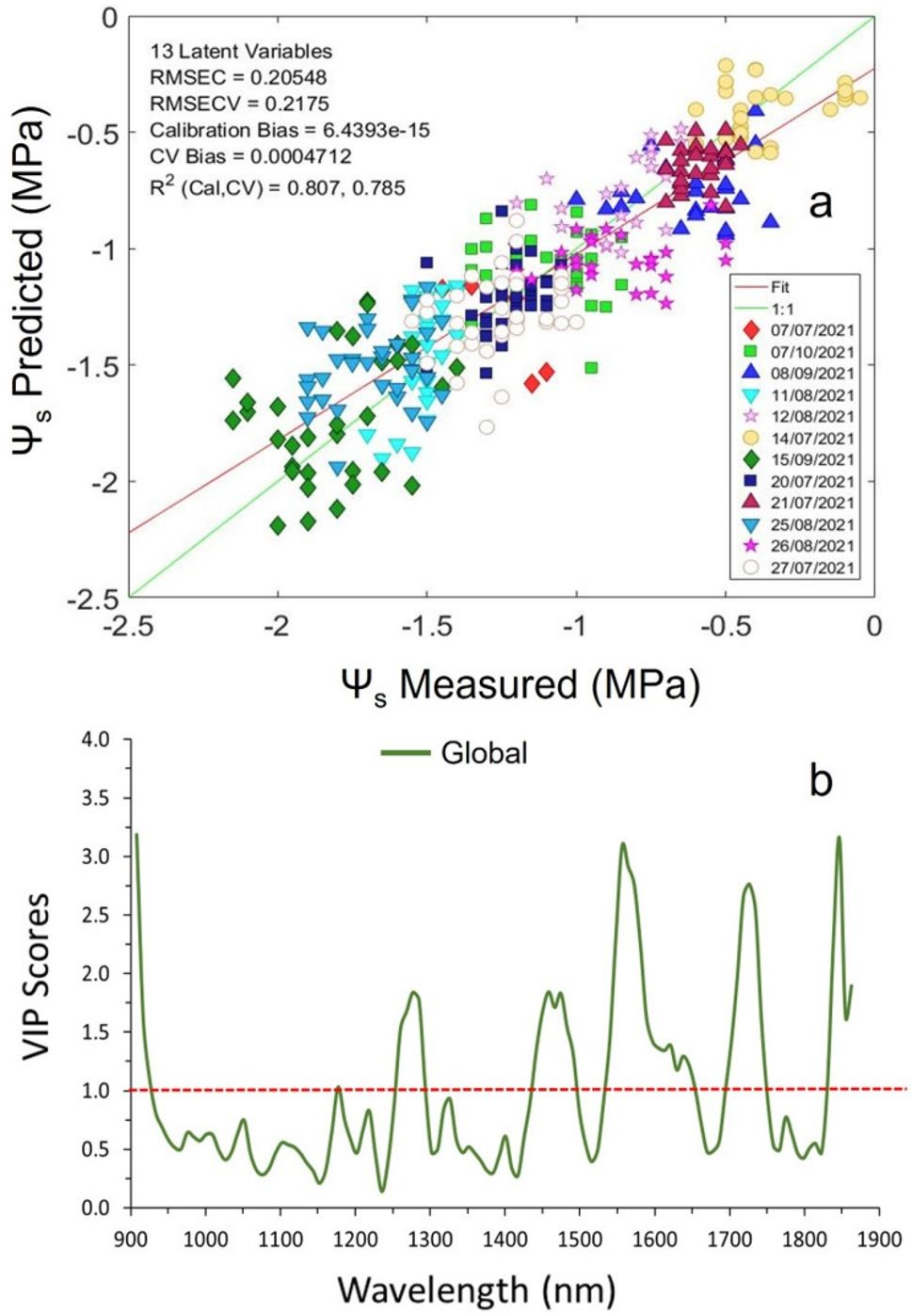
(**a**) The best PLS regression global model for Ψ_s_ prediction using sensor #2 (1.7 NT/H spectrometer) in the Tempranillo and Graciano commercial vineyards during 2021 season and (**b**) Variable Importance in the Projection (VIP) score value for the PLS model.

In order to assess the generalization capability of the models to predict Ψ_s_ with regard to the grapevine variety, a global model involving all data from the two grapevine varieties acquired with sensor #2 was built. The best regression model for Ψ_s_ yielded a R^2^_cv_ value of 0.78, a RMSECV of 0.217 MPa and thirteen latent variables were used without causing an overfitting in the PLS model (Fig. 6a). The most relevant wavelengths of the global PLS model to estimate the water status of the plant using both varieties together were fairly consistent compared to the individual ones (Fig. 6b).

Additional regions in terms of VIP scores were observed at 1548–1580, 1718–1734 and 1268–1285 nm, which are associated to the first N–H stretch overtone (-CONH), to the first C–H stretch overtone (CH2) and the second C–H stretch overtone (C–H), respectively^29^. While these bonds are certainly not related to the water molecule, they may refer to other compounds, such as amino acids (e.g. proline) and other metabolites (e.g. abscisic acid) present in the grapevine leaves that could respond to the presence/absence of plant water stress^30^. Nevertheless, this hypothesis should be confirmed with further physiological experimentation. Moreover, as water stress increases and leaf water content diminishes, the effect of dry leaf matter on spectral reflectance becomes more important^31,32^. In fact, relevant regions in the estimation of Ψ_s_ in the NIR range between (1520–1540 nm)^33^ and (1650–1850) were also reported^31^.

**Figure 7:**
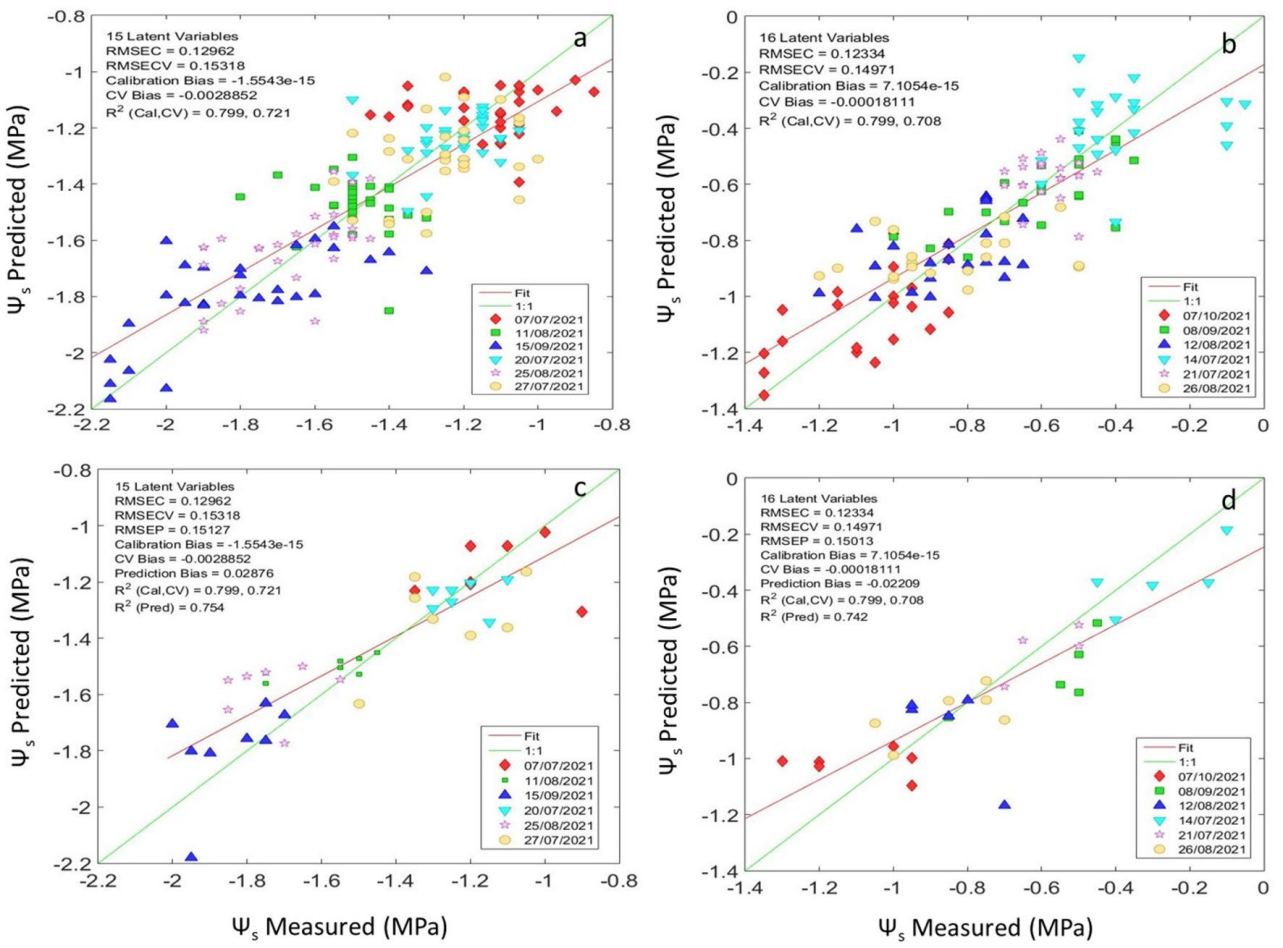
The best PLS regression model for cross validation (**a**,**b**) and external validation (**c**,**d**) to estimate the Ψs using sensor #2 (1.7 NT/H spectrometer) in (**a**) a Tempranillo and (**b**) Graciano commercial vineyards during 2021 season.

For a more realistic evaluation of PLS models’ performance, the original data set was separated in two different subsets to estimate the stem water potential in the Tempranillo and Graciano commercial vineyards (Fig. 7).

The R^2^_p_ and RMSEP values obtained with sensor #2 for Tempranillo variety (R^2^_p_ = 0.75 and RMSEP = 0.151 MPa) and Graciano variety (R^2^_p_ = 0.74 and RMSEP = 0.150 MPa) were very similar using 15 and 16 latent variables according to the criteria mentioned above. These results, compared with the original data set (Fig. 5) showed improved performance for the two varieties (R^2^_cv_ = 0.72; RMSECV = 0.153 MPa in Tempranillo, and R^2^_cv_ = 0.71; RMSECV = 0.149 in Graciano). In addition, it should be highlighted that the results returned for the external validation models with a set of samples not used in the training set provide a reliable estimation for stem water potential in real time to manage irrigation strategies in commercial vineyards.

The micro NIR sensor described in this study (sensor #2) weights 130 g, its dimensions are 108 × 77 × 21 mm, and its cost did not exceed 4.500 € in comparison with other more expensive (> 5 fold) and heavier NIR spectrometers used in this study (sensor #1) and previous works^8,22^, with little reduction in Ψ_s_ estimation performance (in terms of R^2^_cv_ and R^2^_p_) or no changes in the RMSECV and RMSEP, as compared to those reported in these two works. According to Shenk and WesterHaus^34^ values of R^2^_cv_ and R^2^_p_ around 0.70 can be considered as sufficiently reliable to classify and estimate the Ψ_s_ in three different levels (e.g. low, medium and high) and would serve to facilitate the decision-making process about the water use efficiency and the irrigation scheduling in precision viticulture, as three different irrigation scheduling could be defined accordingly.

In comparison to other works, Pampuri et al.^20^ built a PLS-model to predict Ψ_PD_ in *Vitis vinifera* L. Pinot Blanc vines, using Vis/NIR (350–2500 nm) spectra taken on leaves, reaching R^2^_P_ = 0.70 and RMSEP = 0.056 MPa. Using the same spectral range, Cotrozzi et al.^15^ reported lower values of R^2^_P_ (0.44–0.51) with RMSEP between 0.01 and 0.09 MPa, when Ψ_PD_ was estimated from spectral measurements acquired using a leaf-clip assembly on the adaxial side of *Quercus oleoides*. In grapevines, the range of Ψ_PD_ (usually > − 0.2 MPa to < − 0.8 MPa) is much smaller than that of Ψ_S_ (> − 0.6 MPa to < − 1.4 MPa) (Mirás-Avalos and Araujo 2021). ^35^Therefore, the values of RMSEP in these works and that of the present study for the PLS model (RMSEP ~ 0.151 MPa for both varieties) using the whole spectrum are very similar. A very comprehensive work^32^ compared 38 different pipelines (involving several spectral data groups, processing techniques, linear and machine-learning regression models, etc.) for estimating grapevine Ψ_s_ in two commercial vineyards of New Zealand, using spectral data acquired again with the same hand-held spectrophotometer operating in the whole Vis/NIR range (350–2500 nm). The best predictive performance (R^2^_P_ = 0.85, RMSEP = 0.110 MPa) was obtained applying PLS to simple ratio indices. This simplified approach was also attempted by other authors who reported high accuracy (R^2^ ~ 0.80) in quantification^36^ and classification of grapevine water status^37^ based on Ψ_PD_ in different Portuguese cultivars (e.g. Touriga Nacional, Touriga Franca and Tinta Barroca) using several vegetation indices (Visible Atmospherically Resistant Index, VARI; Normalized Difference Greenness Vegetation Index, NGVI; Normalized Reflectance Index, NRI_554, 561_ and the Water Index, WI_900, 970_) computed from spectral data acquired manually with a handheld spectroradiometer covering the Vis/NIR range between 400–1000 nm. In a more recent work of the same research group^38^ the authors used a self-learning artificial intelligence algorithm to analyze the spectral data gathered in the previous studies and reported R^2^ of cross validation higher than 0.95 between estimated and measured Ψ_PD_ values. It is worth highlighting that, in contrast to all these works, in which spectral data were manually taken using portable spectrometers, in the present study, spectral measurements were acquired on-the-go, from a moving vehicle, using two different spectral sensors. This continuous spectral monitorization not only enables the acquisition of a large amount of data, spatially distributed along the vineyard plot in a continuous way, but leverages one of the difficulties of this technology to assess vineyard water status, that is the lack of stand-alone sensors^20^.

### Mapping vineyard water status variability

**Figure 8:**
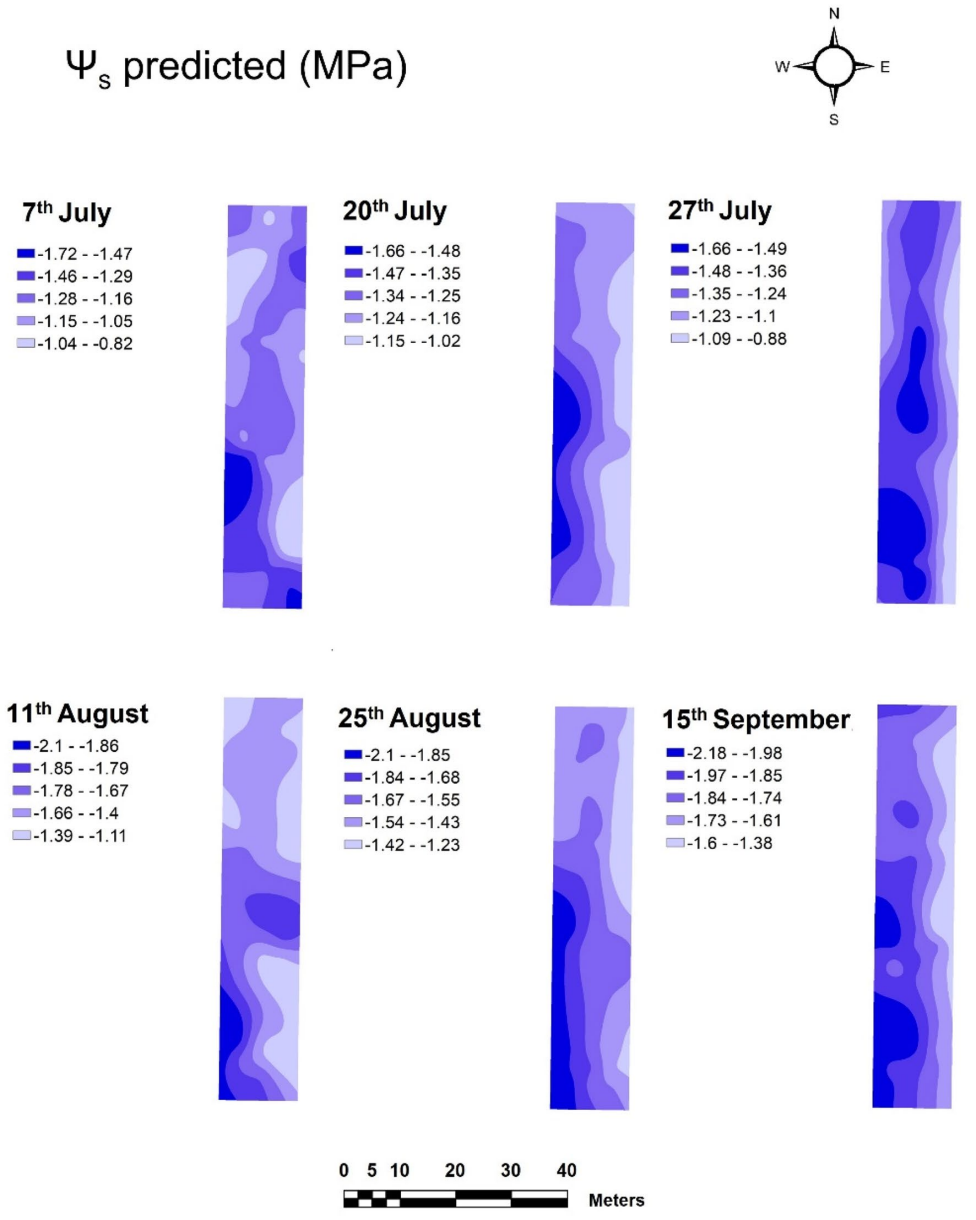
Prediction maps of the spatial variability of Ψ_s_ generated from the PLS model built from on-the-go spectral measurements acquired with sensor #2 mounted on a ground vehicle in a commercial Tempranillo vineyard during season 2021.

**Figure 9:**
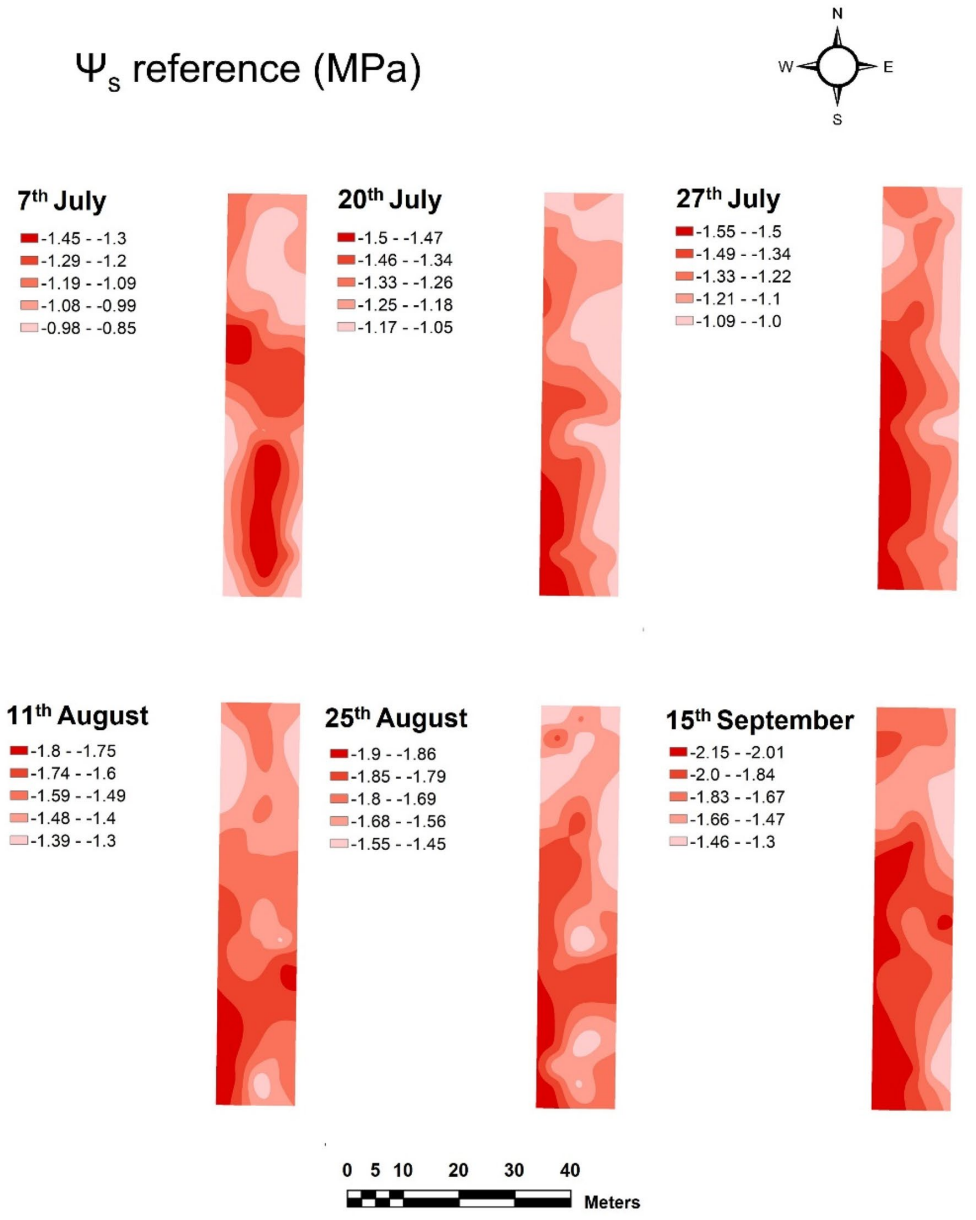
Maps of the spatial variability of Ψ_s_ measured with the reference method in a commercial Tempranillo vineyard during season 2021.

The spatial variability of the vineyard water status acquired with the NIR micro spectrometer (sensor #2) was computed and presented as maps from the predicted values of Ψ_s_ obtained with the ground vehicle and collected with the reference method (Schölander pressure bomb) in the Tempranillo (Figs. 8, 9) and Graciano (Figs. S3 and S4) commercial vineyards. The six sampling dates gathered per grapevine variety were represented to assess the evolution of the vineyard water status along the whole season.

#### Spatial variability of stem water potential in the vineyard plots

The prediction maps of the spatial variability of stem water potential (Ψ_s_) in the Tempranillo plot are displayed in Fig. 8. The most stressed vines (with more negative Ψ_s_ values) were mainly found on the western side and towards the central part of the vineyard plot, while the grapevines on the eastern and slightly north-western side reflected considerably less water stress throughout the period studied. The distribution of the different water stress areas corresponds to the application of the irrigation treatments, described in section “Vineyard site and experimental layout”, during the six dates of the experimental study, reaching the highest values of water stress in the last two dates (Fig. 8).

The spatial variability of the Ψ_s_ provided by the reference method across the six different dates between July 7th and September 15th (Fig. 9) showed very similar distribution to in the estimated values in Fig. 8. The spatial distribution of water stress zones, as well as the stem water potential values taken on each of the measurement dates confirm the good consistency between the map generated from the prediction model (Fig. 8) and the one developed with the reference method (Fig. 9) in the Tempranillo plot. Maps of predicted and reference values of Ψ_s_ for the Graciano’s plot can be found as Figs. S3 and S4 in the supplementary material.

### Development of a custom software for automated data acquisition

With subsequent utilizations of the miniaturized NIR spectral sensor it was becoming clear that the software operation on the computer was a major bottleneck. The software included by the manufacturer was intended for laboratory conditions, with limited features, and it was not adequate for devices with small screens or touch controls which sometimes are the only options available in field operations.

**Figure 10:**
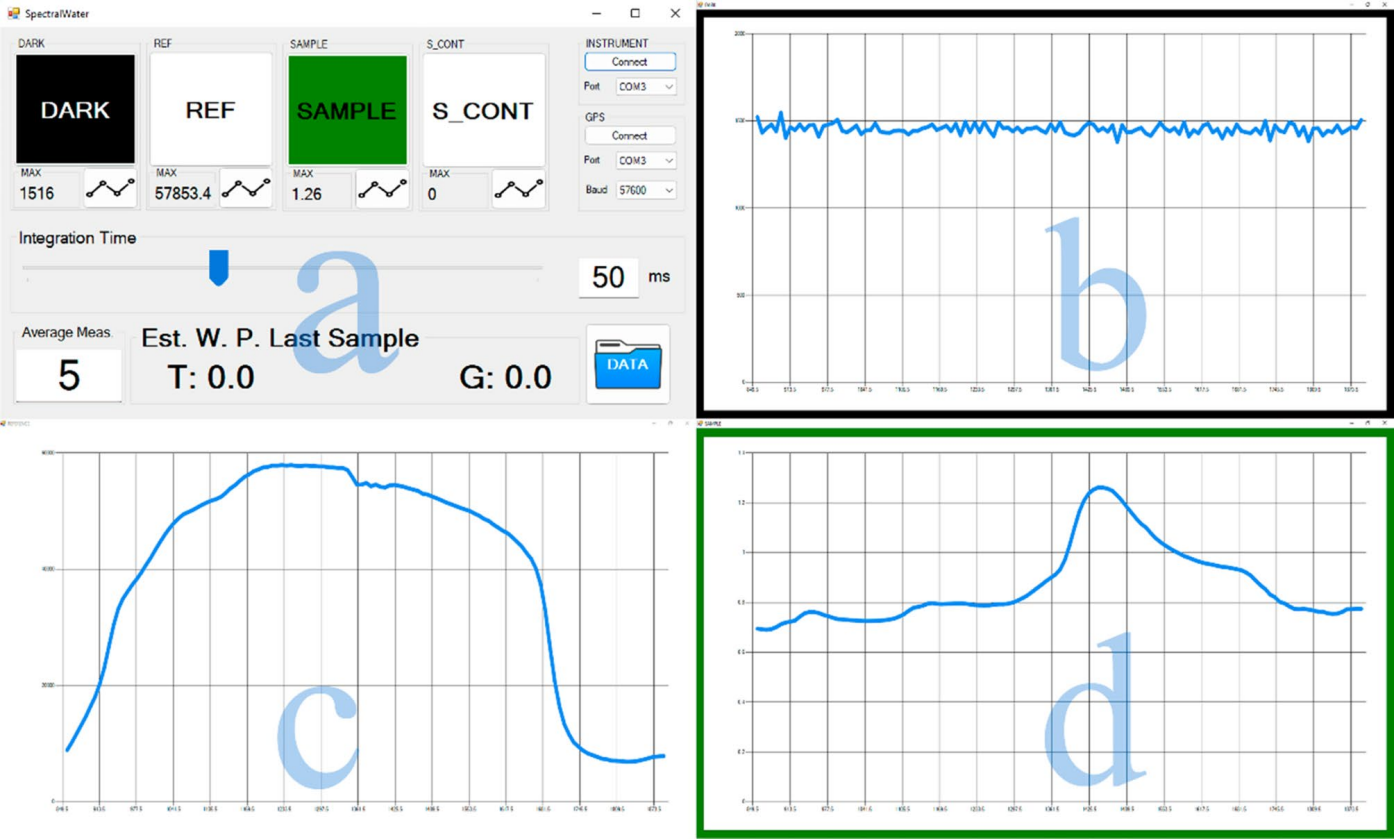
Examples of (**a**) Screenshot of the developed software for automated spectra acquisition with sensor #2; (**b**) dark reference spectrum; (**c**) white reference spectrum; (**d**) leaf signature spectrum.

To mitigate these limitations, it became mandatory to develop a new piece of software specially tailored for taking measures of vine plants in the field under the conditions of the experiment. The user interface of the software was kept as simple as possible, exposing only the needed controls for our usage, as well as big buttons for the main operations and a slider for the integration time that made the software user friendly even on small screens (Fig. 10a). The process of saving the spectra was completely automated to reduce the possibility of a human error in the field to a minimum.

Usage flow of the software was expected to be performed pressing the buttons from left to right. First, the dark reference was taken (Fig. 10b), with the collimator covered. This allowed for temperature compensation and eliminating a big part of the measuring noise coming from the sensor. The next button took the white reference, using a piece of Spectralon material placed at the same distance as the vegetative wall, usually just before starting the measurements with the vehicle ready (Fig. 10c). The third button took the measurement of a single sample corresponding to a healthy leaf, namely leaf spectral signature (Fig. 10d). The last button started and stopped the measurements, saving a final result file with the timestamp that contained only the spectra that were similar to the single leaf measurement (leaf spectral signature) after automated cosine similarity filtering, to avoid spectral interferences due to existing holes and other canopy elements of the canopy (Fig. 10a). This control turns red when a measurement is taking place to clearly make the user aware of the situation. Note that a background colour related to the value being represented is used in each of the plots (Fig. 10b–d) so the user always knows which item is being represented even if the conditions make the screen text difficult to read.

Note that a smaller button and a text label showing the average of the 5 biggest values were added for each one of these steps (Fig. 10a). The button shows a plot for the respective spectra, and the label allows the user to perform a sanity check immediately. Finally, a row was added in the lower part of the window to show the predicted water potential using the latest available model for each of the plots. An individual file gets generated in each step, to allow for future analysis.

Each measurement was also automatically georeferenced using a centimetric RTK GPS system, adding the possibility to import the raw data to a geographic information software. The software includes the functionality to establish connection with the GPS and the instrument.

The first field tests with the newly developed software were already showing major time saves, that accounted for up to 40% of the time spent performing the measurements once the field technicians became acquainted with the software. These improvements came both from the more agile preparations before starting a measurement, and also from the much more infrequent repetitions due human errors. In the case a repetition was needed, (e.g. occurrence of a sudden and drastic change in lightning conditions), the time losses were also minimized.

## Future work and applications

It would have been desirable to implement some other features oriented to further strengthen the capabilities of our software and to ease even more the data acquisition process. An example of these future improvements involves the inclusion of an automated trigger, based on GPS position, as an alternative to the manual approach used in the current version of the software. More stability and user-friendliness focused changes are also expected to be included in the next software iteration, taking advantage of the obtained experience gathered while performing these experiments.

The promising outcomes yielded with this miniaturized NIR spectral sensor reveal the need for further studies considering more grapevine cultivars, seasons and locations to maximise the vineyard water status variability and enhancing their accuracy, robustness and reliability of the predictive models, in the context of precision and sustainable viticulture.

Once refined, this method can be used in the vineyard with minor adjustments to provide a real-time measure of the stem water potential of grapevines, and its spatial variability within a vineyard, to support decision making regarding differential irrigation management to match subzones in the vineyard of i.e. low, medium and high vine water stress, enabling water and cost savings. In other words, the developed monitoring solution, based on semi-automated NIR spectroscopy using a low-cost, simple sensor could provide useful and relevant information of grapevine water status and its variability within the vineyard towards increased sustainability of irrigation, therefore of grapegrowing.

## Conclusions

The NIRS prediction models developed for the determination of the stem water potential (Ψ_s_) using the miniaturized spectral sensor (sensor #2) presented a predictive capacity very similar to those collected by a larger and heavier sensor (sensor #1) during the monitoring of the plant water status in two commercial vineyards. Therefore, this miniaturized, low-cost spectral sensor shows a great potential to become a reliable monitoring tool to assess vineyard water status and estimate its spatial variability in different commercial vineyards with the ability to replace the tedious and time-consuming conventional techniques, providing useful information for a better irrigation scheduling.

Knowledge of spatial variability together with the ability to monitor the temporal evolution of the plant's water status through visualization tools such as heat maps can help define and program precise irrigation strategies during the different phenological stages of the vine, especially in view of the more efficient and sustainable management of irrigation in precision agriculture.

The advancement towards automation of the spectral acquisition and pre-processing presented in this work mitigates one of the major weaknesses and threats of Vis/NIR spectroscopy to become an operative solution for vineyard water status assessment to drive irrigation scheduling in the short term in the wine industry worldwide.

### Supplementary Information

Supplementary Figure S1.

Supplementary Figure S2.

Supplementary Figure S3.

Supplementary Figure S4.

## Data Availability

The datasets used and/or analysed during the current study available from the corresponding author on reasonable request.

## List of symbols

Ψ_s_: Stem water potential
g_s_: Stomatal conductance
InGaAs: Indium gallium arsenide
LV: Latent variable
NIR: Near-infrared
NIRS: Near-infrared spectroscopy
PLS: Partial least squares
R^2^_c_: Determination coefficient of calibration
R^2^_cv_: Determination coefficient of cross validation
RMSEC: Standard error of calibration
RMSECV: Standard error of cross validation
SD: Standard deviation
SNV: Standard normal variate
VIP: Variable importance in the projection
