# Supplementary material for: Towards the automation of NIR spectroscopy to assess vineyard water status spatial–temporal variability from a ground moving vehicle
Source: Sci Rep. 2023 Aug 17;13:13362. doi: 10.1038/s41598-023-39039-z (PMC10435444; doi:10.1038/s41598-023-39039-z)
Supplement: Supplementary file 1 — Supplementary Figure S1. [file 41598_2023_39039_MOESM1_ESM.docx]

**
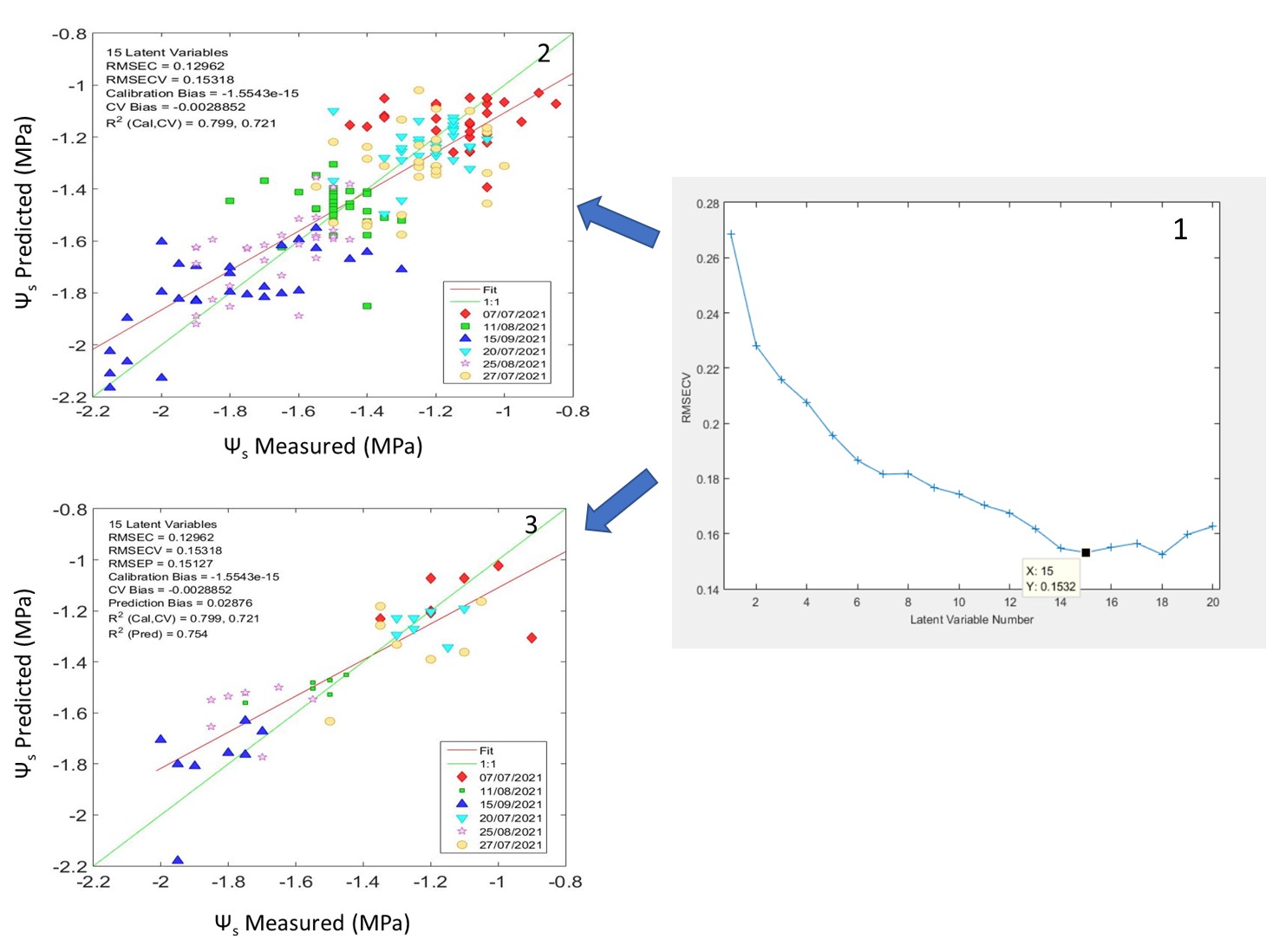
**

**Figure S1.** Latent variables selection using the error curve (1) to predict the Ψ_s_ with the dataset of cross validation (2) and external validation (3) in the Tempranillo vineyards during 2021 season.
