# Supplementary material for: Towards the automation of NIR spectroscopy to assess vineyard water status spatial–temporal variability from a ground moving vehicle
Source: Sci Rep. 2023 Aug 17;13:13362. doi: 10.1038/s41598-023-39039-z (PMC10435444; doi:10.1038/s41598-023-39039-z)
Supplement: Supplementary file 2 — Supplementary Figure S2. [file 41598_2023_39039_MOESM2_ESM.docx]

**
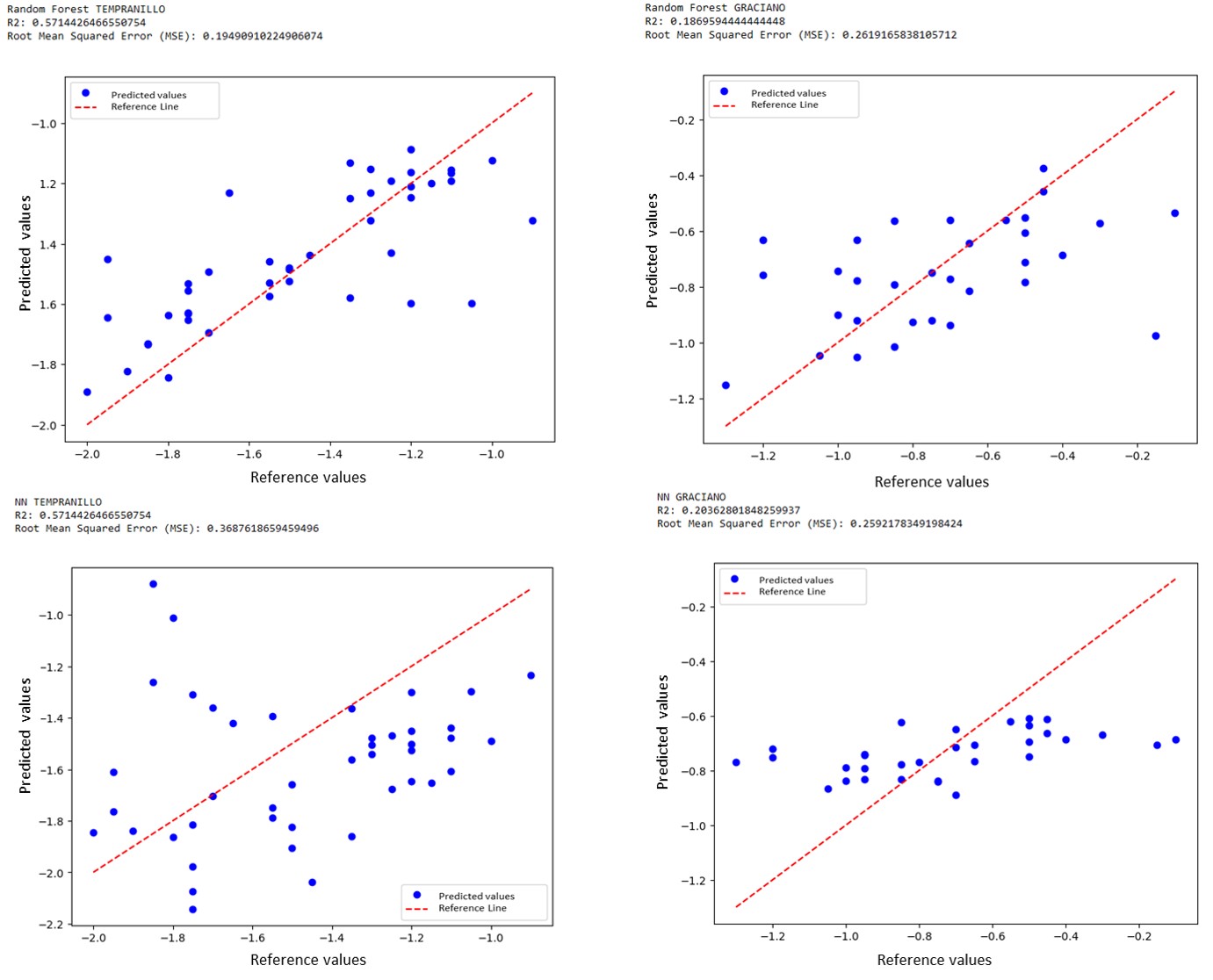
Figure S2.** The best machine learning models applying a Random Forest Regressor and a Multi-layer Perceptron Regressor for Ψ_s_ prediction using sensor #2 (1.7 NT/H spectrometer) in Tempranillo and Graciano during 2021 season.
