# Supplementary material for: Towards the automation of NIR spectroscopy to assess vineyard water status spatial–temporal variability from a ground moving vehicle
Source: Sci Rep. 2023 Aug 17;13:13362. doi: 10.1038/s41598-023-39039-z (PMC10435444; doi:10.1038/s41598-023-39039-z)
Supplement: Supplementary file 3 — Supplementary Figure S3. [file 41598_2023_39039_MOESM3_ESM.docx]

**
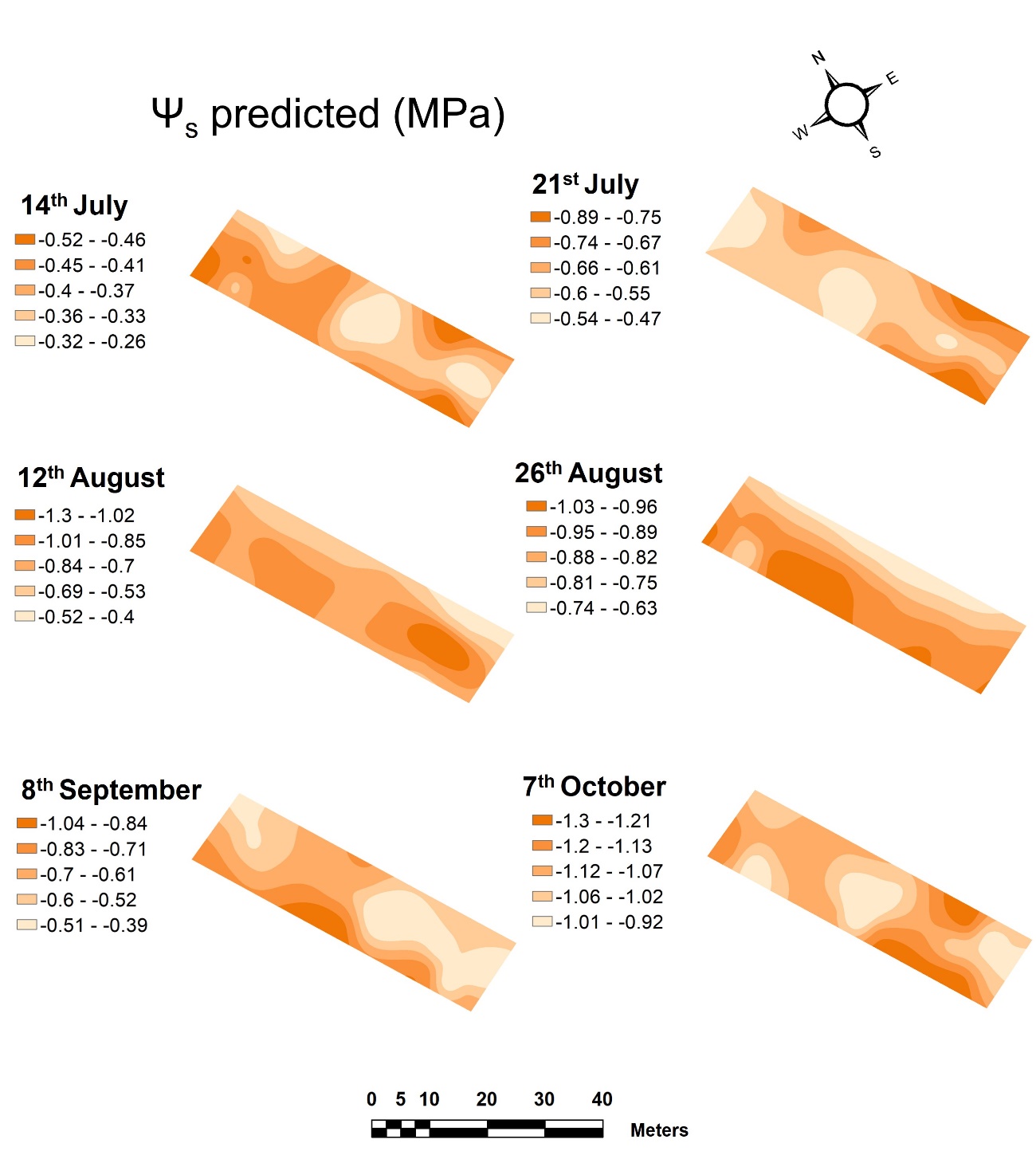
**

**Figure S3** Prediction maps of the spatial variability of Ψ_s_ generated from the PLS model built from on-the-go spectral measurements acquired with sensor #2 mounted on a ground vehicle in a commercial Graciano vineyard during season 2021
